# Reprogramming the aging ovarian microenvironment via mitochondrial sharing and structural remodeling

**DOI:** 10.7150/thno.119957

**Published:** 2025-08-16

**Authors:** Chia-Jung Li, Li-Te Lin, Pei-Hsuan Lin, Jim Jinn-Chyuan Sheu, Zhi-Hong Wen, Kuan-Hao Tsui

**Affiliations:** 1 Department of Obstetrics and Gynecology, Kaohsiung Veterans General Hospital, Kaohsiung 813, Taiwan.; 2 Institute of Biopharmaceutical Sciences, National Sun Yat-sen University, Kaohsiung 804, Taiwan.; 3 Center of General Education, Cheng Shiu University, Kaohsiung 833, Taiwan.; 4 Center of General Education, Shu-Zen Junior College of Medicine and Management, Kaohsiung 821, Taiwan.; 5 National Museum of Marine Biology & Aquarium, Pingtung 944, Taiwan.; 6 School of Medicine, College of Medicine, National Sun Yat-sen University, Kaohsiung 804, Taiwan.; 7 Department of Obstetrics and Gynecology, National Yang-Ming University School of Medicine, Taipei 112, Taiwan.; 8 Department of Obstetrics and Gynecology, Taipei Veterans General Hospital, Taipei 112, Taiwan.; 9 Institute of Biomedical Sciences, National Sun Yat-Sen University, Kaohsiung 804201, Taiwan.; 10 Department of Marine Biotechnology and Resources, National Sun Yat-sen University, Kaohsiung 804, Taiwan.; 11 Department of Medicine, Tri-Service General Hospital, National Defense Medical Center, Taipei 114, Taiwan.

**Keywords:** Ovarian aging, Mitochondrial transfer, Cell-cell communication, Reproductive microenvironment

## Abstract

**Rationale:** Mitochondrial dysfunction in ovarian granulosa cells (GCs) and cumulus cells (CCs) is a defining feature of reproductive aging, contributing to impaired oocyte quality and reduced fertility. This study investigates whether enhancing cytoskeletal dynamics or promoting structural contact between cells can restore mitochondrial function and mitigate ovarian aging.

**Methods:** Mitochondrial exchange was assessed using co-culture systems, live-cell imaging, and mitochondrial labeling in human ovarian somatic cells. Cytoskeletal modulation was achieved using FTY720, and cell-cell contact was enhanced through soft 3D extracellular matrix (ECM) scaffolds. Functional outcomes were evaluated through ATP assays, mitochondrial membrane potential, Seahorse bioenergetics profiling, and transcriptomic analysis. In vivo validation was conducted in aged mice treated with FTY720.

**Results:** Granulosa and cumulus cells exchanged mitochondria via tunneling nanotubes (TNTs), a process significantly reduced with age. Mitochondrial transfer was contact-dependent and not mediated by paracrine signaling. FTY720 enhanced TNT formation and mitochondrial delivery, restoring ATP levels, membrane potential, and oxidative phosphorylation in aged cells. 3D ECM culture promoted spheroid formation, activated YAP signaling, and improved mitochondrial function without pharmacological agents. In aged mice, FTY720 treatment increased follicle numbers, improved oocyte mitochondrial quality, and elevated serum AMH levels.

**Conclusions:** These findings demonstrate that somatic cell contact is essential for mitochondrial complementation in aging ovaries. By promoting intercellular connectivity through cytoskeletal or microenvironmental remodeling, endogenous mitochondrial sharing can be reactivated to restore bioenergetic function. This approach offers a novel regenerative strategy to counteract reproductive aging.

## Introduction

Female reproductive aging is characterized by a progressive decline in oocyte quality and developmental competence, leading to reduced fertility, increased miscarriage rates, and a higher risk of chromosomal abnormalities 1, 2. This deterioration is driven not only by intrinsic oocyte damage, such as spindle instability and mitochondrial dysfunction, but also by age-related changes in the surrounding somatic follicular environment, including CCs, GCs, ECM, and local signaling factors 3, 4. As the primary cellular niche supporting the oocyte, CCs and GCs play essential roles in nutrient delivery, metabolic cooperation, and meiotic regulation 5. A growing body of evidence suggests that mitochondrial dysfunction is central to reproductive aging. In oocytes, GCs, and CCs, aging is linked to diminished mitochondrial membrane potential, lower ATP production, and increased accumulation of reactive oxygen species (ROS) 6, 7. Mitochondria not only fuel oocyte maturation but also serve as critical regulators of redox balance and apoptotic signaling 8. Interventions targeting mitochondrial function, such as coenzyme Q10 or NAD^+^ precursors, have shown potential in animal models for restoring bioenergetic capacity and improving fertility outcomes 9, 10.

GCs and CCs intimately interact with the oocyte through transzonal projections (TZPs) and connexin-based gap junctions, forming a highly coordinated soma-germline communication network 11, 12. Through these channels, CCs and GCs supply pyruvate, amino acids, ions, and regulatory molecules to the oocyte, facilitating proper meiotic progression and cytoplasmic maturation 13. However, with age, both the number and function of TZPs decline, compromising the oocyte's access to somatic support 14, 15. GC apoptosis and altered gene expression profiles further contribute to follicular atresia and reproductive failure 16. The ovarian microenvironment possesses significant rejuvenating potential. When aged oocytes are transferred into a youthful somatic microenvironment, notable improvements are observed in their meiotic capacity and mitochondrial activity. This shift also leads to a reduction in oxidative stress, allowing some oocytes to progress to the blastocyst stage and even result in live births 17. This compelling evidence highlights the critical role of somatic cell derived support in maintaining oocyte health and suggests that reconstructing a youthful microenvironment could effectively reverse certain hallmarks of reproductive aging.

Similar mechanisms of organelle sharing have been observed in other biological systems. Intercellular mitochondrial transfer through TNTs, which are projections composed of filamentous actin, has been documented in lung epithelial cells, neurons, and immune cells as a response to cellular stress 18-21. These transfers can restore ATP production and decrease ROS levels in recipient cells that are metabolically compromised. Although well characterized in non-reproductive tissues, the relevance of this process in the ovary remains largely unexplored. Emerging evidence from other organ systems suggests that mitochondrial donation plays a pivotal role in cellular repair and rejuvenation. For example, mesenchymal stem cell (MSC)-mediated mitochondrial transfer has been shown to rescue senescent or stressed recipient cells through TNT-like structures, leading to improved energy metabolism and cellular homeostasis 22-24. Such mechanisms could hold translational potential for aging-related diseases, including ovarian insufficiency.

Beyond passive exposure to young somatic cells, therapeutic strategies to counter ovarian aging have expanded to include mitochondrial transplantation, ooplasmic transfer, and pharmacological stimulation of mitochondrial biogenesis 25, 26. However, these approaches are often constrained by invasiveness, ethical considerations, and inconsistent efficacy. An alternative strategy may lie in modulating the intrinsic cytoskeletal architecture of aging follicles to facilitate organelle sharing and maintain signaling integrity within the cumulus-oocyte complex.

To evaluate whether mitochondrial rescue can be achieved through structural and physical enhancement of intercellular connectivity, we combined two complementary interventions: pharmacologic cytoskeletal activation and microenvironmental remodeling. FTY720, a sphingosine-1-phosphate receptor modulator, was chosen for its reported capacity to promote TNT formation with low cytotoxicity. Concurrently, we employed tunable 3D ECM hydrogels with physiologically relevant stiffness, designed to mimic the ovarian cortex and restore native granulosa cell architecture. Together, these strategies aim to potentiate soma-to-soma mitochondrial exchange and energy flow. Unlike invasive mitochondrial replacement or systemic metabolic supplementation, this approach leverages endogenous cellular cooperation, providing a structurally grounded mechanism to restore mitochondrial integrity in aging ovarian tissue.

## Materials and Methods

### Granulosa Cell Culture

Human ovarian granulosa cells (HGL5; Applied Biological Materials Inc.) were maintained in DMEM/F12K medium supplemented with 10% fetal bovine serum (FBS), 1% penicillin-streptomycin, 2% Ultroser G (Pall Corp.), and 1% ITS Plus (Zen-Bio). Cells were cultured at 37 °C in a humidified incubator with 5% CO₂. Early-passage cells (< P10) were designated as “young,” while late-passage cells (> P60) were classified as “aged.” These thresholds were adopted based on our previous publication 27, which demonstrated that HGL5 cells at high passage exhibit mitochondrial dysfunction, elevated oxidative stress, and increased expression of aging markers such as p16 and p21. This system provides a consistent and validated *in vitro* model for studying granulosa cell aging.

### Mitochondrial Functional Analysis

Following treatment, cells were washed, resuspended in culture medium, and stained with DCFDA (10 μM), MitoSOX (5 μM), TMRE (500 nM), MitoTracker green (250 nM, Life Technologies, CA, USA) and ATP (BioTracker ATP-Red Live Cell Dye, Merck, SCT045, 10 μM ) at 37 °C for 30 minutes. After washing, samples were analyzed by flow cytometry (CytoFLEXTM, Beckman Coulter, CA, USA).

### Mitochondrial Transfer Assay and Live Imaging

Mitochondrial transfer was assessed by dual labeling with MitoTracker Green and MitoTracker Red. After 24 h of co-culture, fluorescence colocalization was used to identify organelle exchange. Time-lapse imaging was performed using the NanoLive 3D Cell Explorer, with images acquired every 2-5 min over a 4-h period to monitor mitochondrial movement between cells. Data were processed using NanoLive software.

### RNA Extraction and Real-Time PCR

Total RNA was extracted from collected samples using QIAZOL reagent (QIAGEN), followed by cDNA synthesis utilizing the ToolScript MMLV RT Kit with TOOLS M-MLV RTase on the StepOnePlus real-time PCR system (Applied Biosystems). Primer sequences used in real-time PCR are listed in Table S1.

### Western Blot Analysis

Protein samples were separated by SDS-PAGE, transferred to PVDF membranes, blocked, and incubated overnight at 4 °C with primary antibodies (Table S2). Following incubation with HRP-conjugated secondary antibodies, proteins were visualized using enhanced chemiluminescence (ECL, GE Healthcare). Band intensities were quantified using ImageJ software.

### Oxygen Consumption Rate Measurement

Oxygen consumption rate (OCR) was measured using the Seahorse XF HS mini extracellular flux analyzer (Agilent Technologies). Briefly, cells were seeded at approximately 2×10^4^ cells per well, and mitochondrial respiration was monitored continuously. OCR was sequentially assessed after adding oligomycin, FCCP, and antimycin A plus rotenone, according to the previously described protocol 28.

### Metabolomic Analysis and Lactate Measurement

Cellular metabolites were extracted using methanol/chloroform/water extraction and analyzed by ultra-high performance liquid chromatography-tandem mass spectrometry (UHPLC-MS/MS) at the National Taiwan University Centers of Genomics and Precision Medicine, following the protocols described by Liao et al. 29. Heatmaps of central carbon metabolism were generated using MetaboAnalyst. Lactate levels in culture medium were quantified using a Lactate Colorimetric Assay Kit (BioVision).

### 3D Culture and ECM Stiffness Analysis

The granulosa cells were embedded in soft and stiff ECM hydrogels (Matrigen) to establish 3D culture conditions. The mechanical properties of the matrices were validated using atomic force microscope to confirm their rheological stiffness. Spheroid formation and cellular aggregation were assessed by measuring Feret diameter and evaluating cell-cell connectivity.

### Animals

Young (< 10 weeks) and aged (> 35 weeks) female BALB/c mice were purchased from the National Laboratory Animal Center (Taipei, Taiwan). Animals were housed at 25 ± 2°C under a 12-hour light/dark cycle with ad libitum access to food and water. All animal procedures were approved by the Institutional Animal Care and Use Committee (IACUC) of Kaohsiung Veterans General Hospital (approval #2508-2807-25002-NSTC). Each experimental group consisted of 8 female mice (> 40 weeks old), a sample size based on previous work in ovarian aging models yielding consistent and interpretable results.

### *In Vivo* FTY720 Treatment and Ovarian Analysis

Eight-week-old female C57BL/6 mice were aged to 30 weeks and treated with FTY720 (2 mg/kg/week, i.p.) for 8 weeks. Ovaries were collected for histological assessment (H&E), follicle staging, immunostaining for AMH and Miro1, and RNA-seq. MII oocytes were isolated for assessment of polar body extrusion, mitochondrial functions.

### Immunofluorescence

Tissue sections were fixed with 4% paraformaldehyde, permeabilized with 0.2% Triton X-100, and blocked with 3% bovine serum albumin (BSA). Samples were then incubated overnight at 4 °C with primary antibodies listed in Table S2. After three washes with PBST, samples were incubated with appropriate fluorescence-conjugated secondary antibodies for 30 min at room temperature. Staining was performed using the Fluorescence Multiple Stain Kit (BioTnA, TATS01F, Kaohsiung, Taiwan). Slides were scanned using a fully motorized fluorescence microscope (BX61VS, Olympus Corporation) equipped with high-precision autofocus at 20× magnification. Image visualization and analysis were performed using Olympus software at Li-Tzung Pathology Laboratory (Kaohsiung, Taiwan).

### Collection and Culture of Human CCs

Human cumulus-oocyte complexes (COCs) were collected from patients undergoing oocyte aspiration. Cumulus cells were isolated by hyaluronidase treatment and mechanical dissociation, then resuspended in supplemented medium containing FBS, ITS, and androstenedione. Cells were plated at 2 × 10⁴ cells per well and cultured at 37.5°C in 5% CO₂ for up to 24 hours before experimentation.

### Ethics Statement

This study was approved by the Institutional Review Board of Kaohsiung Veterans General Hospital (KSVGH21-CT1-43), conducted according to approved guidelines, and implemented in compliance with the Declaration of Helsinki.

### Statistical Analysis

Experiments were repeated at least three times, with data presented as mean ± SEM. Fluorescence intensities were quantified using ImageJ (NIH), Zen lite (Carl Zeiss), and MicroP software. Statistical significance was determined by two-way ANOVA using GraphPad Prism 8.0, with *p* < 0.05 considered significant.

## Results

### Cell-Cell Aggregation Enhances Mitochondrial Function in Human Cumulus Cells

**Figure 1:**
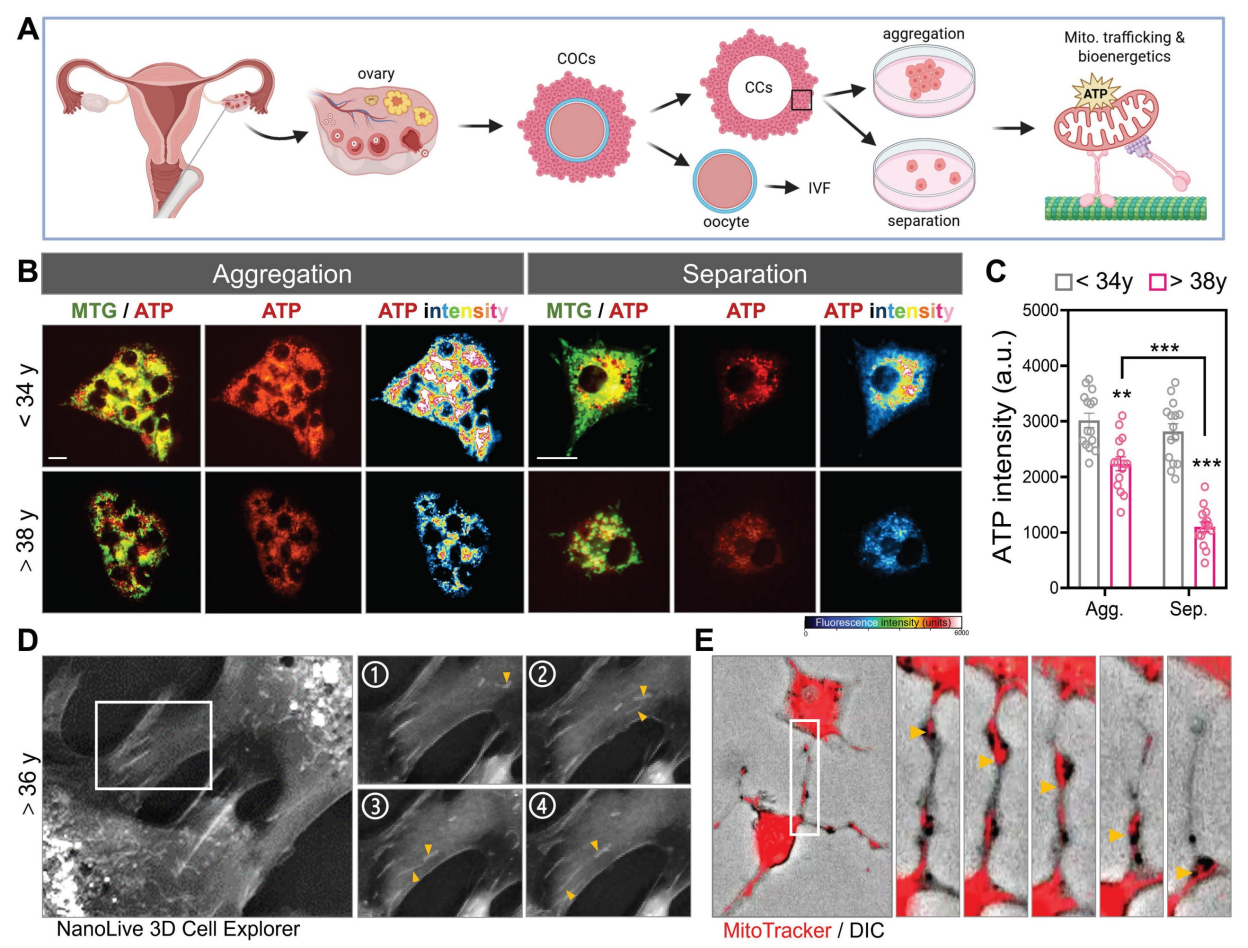
** Human cumulus cell aggregation preserves mitochondrial bioenergetics and enables intercellular mitochondrial transfer.** (A) Schematic representation of mitochondrial energy differences in aggregated versus separated human CCs and GCs. (B) Representative fluorescence images showing mitochondrial morphology (MitoTracker green), ATP signal (red), and pseudocolor ATP intensity heatmaps (blue-red) in CCs from women <34 years and >38 years under two morphological states: mitochondrial aggregation and separation. (C) Quantification of ATP fluorescence intensity (arbitrary units, a.u.) under aggregation (Agg.) and separation (Sep.) states. (D) 3D live-cell imaging using NanoLive in CCs from a patient >36 years old shows long protrusions (boxed region, left) with fragmented mitochondrial ends (yellow arrowheads). Panels 1-4 are sequential time-lapse frames showing dynamic process. (E) MitoTracker (red) and DIC overlay images show intercellular transfer of mitochondria in TNTs (yellow arrows). Scale bar = 20 µm. **p < 0.01, ***p < 0.001.

To investigate whether mitochondrial organization and intercellular connectivity influence metabolic output in human CCs, we examined primary CCs isolated from infertile patients undergoing IVF. Using morphological sorting, cells were categorized into aggregation- or separation-dominant patterns, reflecting the degree of CCs-CCs clustering typically observed in follicular microenvironments. Live-cell staining with MitoTracker Green and an ATP-sensitive fluorescent probe revealed striking differences in bioenergetic capacity. Aggregated CCs exhibited significantly stronger ATP signal intensity than their separated counterparts (Figure 1A-B). Notably, CCs from patients under 34 years displayed robust ATP levels regardless of aggregation state, whereas CCs from patients over 38 years showed a marked reduction in ATP fluorescence, especially under separation conditions. Quantitative analysis confirmed that mitochondrial ATP production was highest in younger, aggregated CCs and lowest in aged, separated cells (Figure 1C), suggesting that both intercellular contact and reproductive age influence the metabolic competence of granulosa-derived somatic cells. To further visualize real-time intercellular organelle dynamics in primary CCs, we employed a label-free optical diffraction-based imaging system that enables high-resolution 3D reconstruction of subcellular architecture in live cells. Based on their morphology and refractive index characteristics, these organelles were consistent with mitochondria. Over the course of 4 consecutive frames, mitochondrial-sized structures (yellow arrowheads) were seen migrating from one CC into its adjacent partner, indicating active intercellular transport (Figure 1D). To directly visualize mitochondrial transfer, we labeled donor CCs with MitoTracker Red and performed high-resolution DIC imaging. Fluorescently labeled mitochondria were observed moving between CCs along TNTs (Figure 1E), confirming that mitochondrial transport occurs spontaneously in human granulosa cell networks.

### Intercellular Mitochondrial Transfer from Young Donors Rejuvenates Aged Granulosa Cells

**Figure 2:**
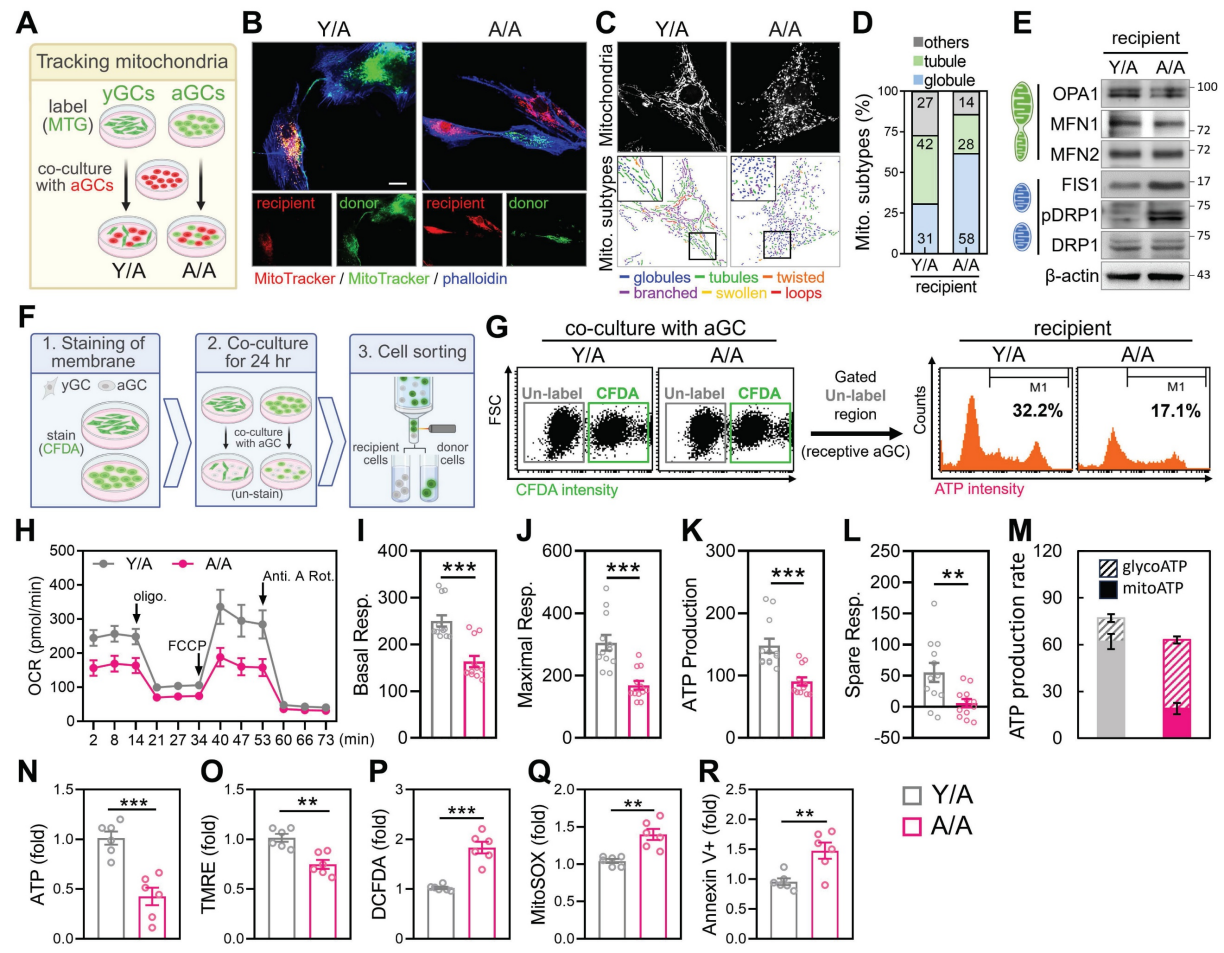
** Mitochondrial transfer from young to aged granulosa cells restores mitochondrial structure and bioenergetics.** (A) Experimental design: young and aged HGL5 cells were labeled with MitoTracker Green and Red, respectively, and co-cultured for 24 h. (B) Representative images of mitochondrial transfer in Y/A and A/A co-cultures (C) Classification of mitochondrial subtypes in recipient cells. Color-coded traces represent distinct mitochondrial morphologies. (D) Quantification of mitochondrial subtypes in recipient aGCs. (E) Western blot analysis of mitochondrial dynamics-related proteins in recipient aGCs. Increased OPA1 and MFN1, and reduced DRP1/pDRP1 are observed in Y/A versus A/A. (F) Workflow for identifying recipient aGCs using CFDA staining and cell sorting. (G) Flow cytometric identification of recipient aGCs and measurement of intracellular ATP intensity (right histogram). Y/A cells display increased ATP signal compared to A/A. (H) Quantification of intracellular ATP levels. (I) TMRE staining indicates improved mitochondrial membrane potential in Y/A group. (J) OCR tracing in recipient aGCs using Seahorse analysis. (K-N) Quantification of basal respiration (K), maximal respiration (L), ATP production (M), and spare respiratory capacity (N). Scale bar = 20 µm. **p < 0.01, ***p < 0.001.

To investigate whether mitochondria from young granulosa cells (yGCs) can functionally restore aged granulosa cells (aGCs), we established an *in vitro* co-culture system using the HGL5 cell line (Figure 2A). Donor cells (either yGCs or aGCs) were first labeled with MitoTracker Green (MTG) and then co-cultured with unlabeled aGCs. Two groups were created: yGCs co-cultured with aGCs (Y/A) and aGCs co-cultured with aGCs (A/A). This setup enabled us to track the directionality of mitochondrial transfer and assess its functional consequences on aged recipient cells (Figure 2A). Long-term passaged HGL5 cells were used to model replicative aGCs, while early-passage cells served as metabolically competent donors. The aged cells displayed classical features of cellular aging, including elevated expression of *p16* and *p21*, along with a marked reduction in ATP levels compared to cells in Y/A co-cultures (Figure S1A-C). MitoTracker Green-labeled yGCs were co-cultured with unlabeled aGCs for 24 hours (Y/A group), and MitoTracker Red imaging revealed clear mitochondrial transfer from donor to recipient cells, which colocalized with F-actin-rich cytoskeletal projections (Figure 2B). Donor-derived mitochondria in recipient aGCs exhibited integration into the host mitochondrial network, suggesting functional incorporation. Morphological quantification revealed a shift in mitochondrial morphology in Y/A cells from globular to more tubular and interconnected forms (Figure 2C-D), a pattern associated with healthier and fusion-prone mitochondria. Western blot analysis further confirmed increased expression of fusion-related proteins and decreased phosphorylation of DRP1, indicating enhanced mitochondrial fusion and reduced fission in co-cultured aged granulosa cells (Figure 2E).

To functionally evaluate whether mitochondrial transfer from young to aged granulosa cells restores metabolic activity, we compared Y/A and A/A co-cultures. Seahorse analysis showed that Y/A co-cultures exhibited higher oxygen consumption rates (OCR) (Figure 2H), with significant increases in basal, maximal respiration, ATP production, and spare respiratory capacity (Figure 2I-L), indicating improved mitochondrial respiratory function in recipient aged cells. Consistent with this, analysis of energy production pathways revealed a higher proportion of mitochondrial-derived ATP and reduced reliance on glycolysis in the Y/A condition (Figure 2M). Flow cytometric staining showed increased total ATP levels (Figure 2N) and higher mitochondrial membrane potential (TMRE) (Figure 2O) in the Y/A group. Moreover, markers of oxidative stress, including hydrogen peroxide (DCFDA) and mitochondrial superoxide (MitoSOX), were significantly lower in Y/A compared to A/A (Figure 2P-Q). Apoptotic cells marked by Annexin V^+^ were also decreased in the Y/A group (Figure 2R), supporting the conclusion that mitochondrial transfer contributes to bioenergetic and survival improvements in aged granulosa cells. To determine whether direct cell-cell contact was necessary for these effects, we used a transwell system allowing soluble factor exchange but preventing physical interaction. In this setup, aged recipient cells showed no improvement in mitochondrial function or ATP production when separated from either young or FTY720-treated donor cells (Figure S1D-F), confirming that physical contact, rather than paracrine signaling, is required for mitochondrial exchange and metabolic restoration.

### Mitochondrial Transfer Reprograms Metabolism in Aged Granulosa Cells

**Figure 3:**
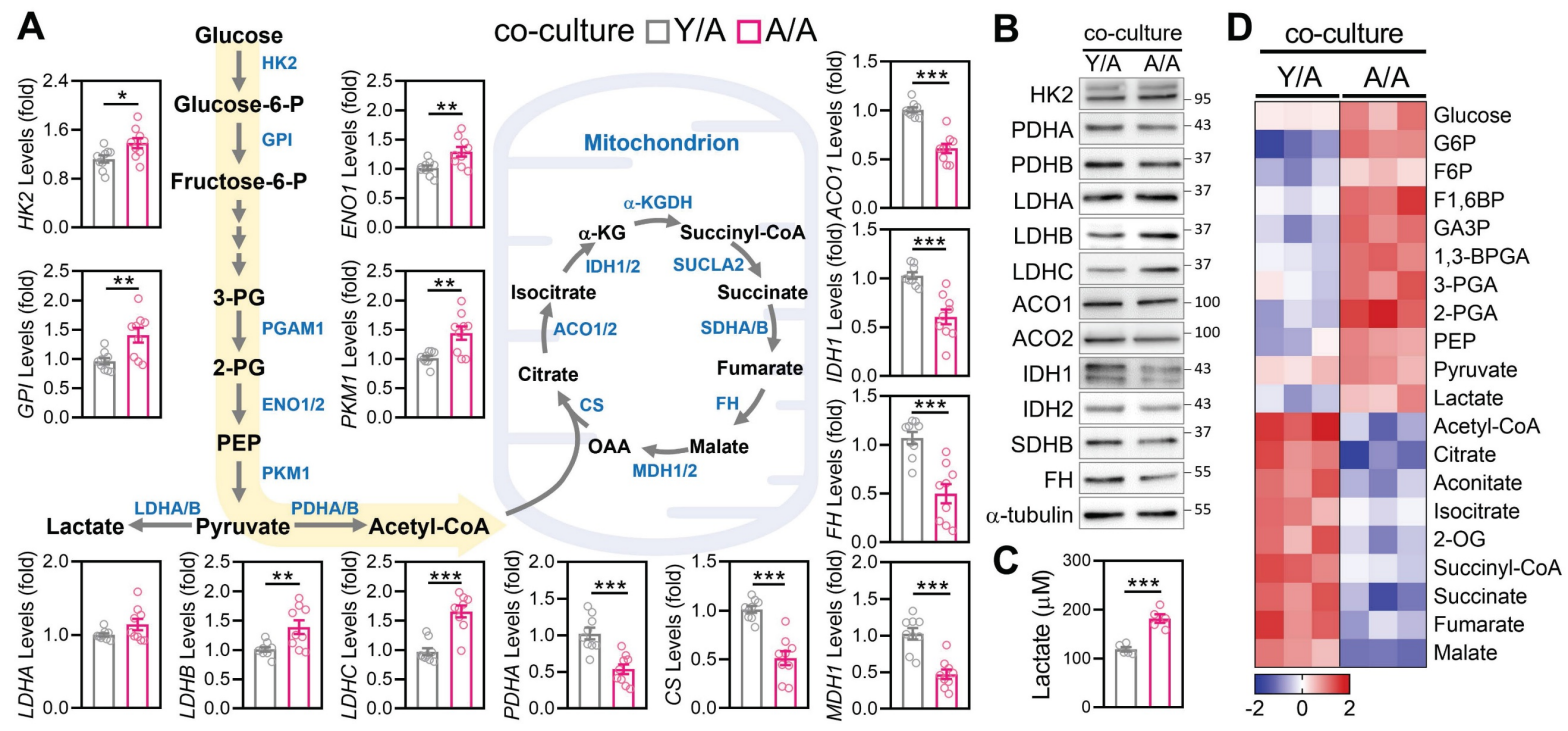
** Co-culture with young granulosa cells reverses metabolic decline in aged cells.** (A) mRNA expression levels of glycolysis and TCA cycle-related genes in aged granulosa cells co-cultured with young cells (Y/A) or aged cells alone (A/A). Genes include *HK2*, *GPI*, *ENO1*, *PKM1*, *LDHA/B/C*, *PDHA*, *CS*, *ACO1/2*, *IDH1*, *FH*, and *MDH1*. (B) Western blot analysis of glycolysis and TCA cycle enzymes confirms reduced expression in A/A recipient cells. α-tubulin serves as the loading control. (C) Measurement of extracellular lactate levels in Y/A and A/A cells. (D) Heatmap of targeted metabolomics analysis showing relative abundance of central carbon metabolites, including glycolytic intermediates, TCA metabolites, and associated energy substrates. Color scale indicates Z-score normalization. *p < 0.05, **p < 0.01, ***p < 0.001.

Given the observed improvements in mitochondrial morphology and ATP production following young-to-aged mitochondrial transfer, we next examined whether this process also restructured core metabolic pathways in recipient aGCs. Transcriptomic profiling revealed that Y/A exhibited significant upregulation of key glycolytic enzymes, including HK2, GPI, ENO1, PKM1, and LDHC, relative to A/A controls (Figure 3A). In parallel, transcripts involved in pyruvate oxidation and the tricarboxylic acid (TCA) cycle—such as PDHA, CS, ACO1, IDH1, FH, and MDH1—were also elevated in Y/A recipients, suggesting a restoration of mitochondrial carbon flux. These gene-level changes were mirrored at the protein level. Western blot analysis confirmed higher expression of HK2, LDHC, ACO1, IDH1/2, and FH in Y/A recipient cells compared to A/A controls (Figure 3B). To determine whether the observed molecular changes corresponded to functional metabolic outcomes, we measured extracellular lactate levels as an indicator of glycolytic activity. A/A cells produced significantly more lactate than Y/A cells (Figure 3C), suggesting a greater reliance on anaerobic glycolysis in the absence of mitochondrial support. Complementary analysis of central carbon metabolism further supported this metabolic reprogramming. Heatmap clustering showed that Y/A recipient cells contained higher levels of tricarboxylic acid (TCA) cycle intermediates, including citrate, alpha-ketoglutarate, succinate, fumarate, and malate, whereas A/A cells accumulated upstream glycolytic metabolites (Figure 3D). This metabolic shift from glycolysis toward oxidative phosphorylation indicates that mitochondrial transfer not only restores mitochondrial structure but also reactivates downstream energy pathways in aged granulosa cells. Collectively, these findings demonstrate that mitochondria donated from young cells promote oxidative metabolism and diminish glycolytic dependence in aged cells, highlighting functional metabolic rejuvenation through intercellular organelle sharing.

### Cytoskeletal Reinforcement Promotes Mitochondrial Turnover Through Intercellular Contact

**Figure 4:**
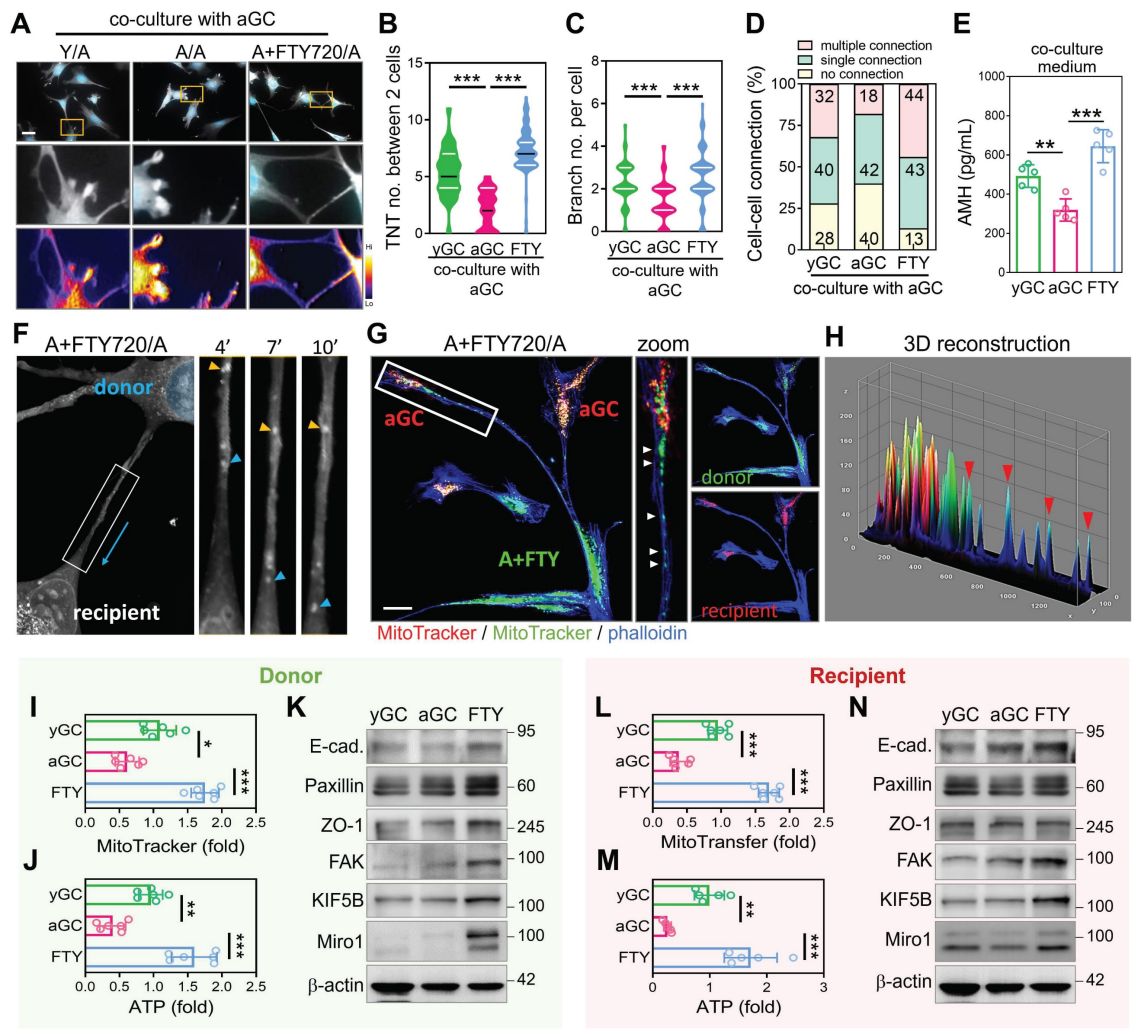
** Cytoskeletal enhancement in aged granulosa cells promotes TNT formation and facilitates mitochondrial energy transfer.** (A) Representative images showing TNT formation between cells in Y/A, A/A, and A+FTY720/A co-cultures. Heatmaps display intensity of TNT structures. (B-C) Quantification of TNT and branch per two cells across groups. (D) Proportions of cell-cell connection types in co-culture settings. (E) ELISA measurement of AMH in co-culture media. (F) Time-lapse imaging of mitochondrial trafficking along TNTs in A+FTY720/A co-cultures. Yellow and blue arrowheads indicate movement of mitochondria. (G-H) Immunofluorescence and 3D reconstruction of mitochondrial transfer from FTY720-pretreated donor cells (green) to untreated aGC recipients (red). Quantification of MitoTracker intensity (I) and ATP levels (J) in donor aGCs after co-culture with FTY720-pretreated donors. (K) Western blotting of cytoskeletal proteins in yGC, aGC, and FTY720-treated aGCs. MitoTracker intensity (L) and ATP levels (M) in recipient cells following donation from FTY-treated cells. (N) Cytoskeletal protein levels in recipient aGCs post-FTY720 transfer. Scale bar = 20 µm. *p<0.05, **p<0.01, ***p<0.001.

While spontaneous mitochondrial transfer between GCs is detectable under native conditions, the frequency and efficiency of this process decline with age. We hypothesized that aGCs may lack the cytoskeletal capacity to form robust TNTs, thereby limiting their ability to engage in mitochondrial exchange. To test this, we pre-treated aGCs with the cytoskeletal enhancer FTY720 for 24 hours, followed by co-culture with untreated aGCs (designated as A+FTY720/A). Immunofluorescence imaging revealed that A+FTY720/A co-cultures formed significantly more TNT-like structures compared to A/A and levels comparable to Y/A (Figure 4A). Quantitative analysis confirmed that the number of TNTs per two cells (Figure 4B), as well as branching complexity per cell (Figure 4C), were both significantly elevated in the FTY720-treated group. Cell-cell connectivity analysis further demonstrated that A+FTY720/A co-cultures exhibited a higher frequency of multiple cellular connections (Figure 4D). Notably, AMH secretion, which serves as a marker of granulosa cell functional competence, was significantly elevated in A plus FTY720 to A co-cultures (Figure 4E). This finding indicates a physiological benefit that extends beyond structural remodeling. To directly visualize intercellular mitochondrial transfer, we conducted time-lapse imaging of aGCs co-cultured with FTY720-pretreated donor cells. As shown in Figure 4F, mitochondria within TNTs could be seen moving unidirectionally from donor to recipient cells over time, as indicated by the blue arrow. To further confirm directional mitochondrial transfer, we labeled FTY720-treated donor cells with MitoTracker Green and aGC recipients with MitoTracker Red. Co-culture imaging revealed that green mitochondria were transferred from a single donor toward two spatially distinct recipient aGCs through branched TNTs (Figure 4G), suggesting efficient and multitarget delivery. Three-dimensional surface reconstruction of the contact regions confirmed co-localization of donor-derived mitochondria within recipient cells (Figure 4H), providing further evidence that mitochondria physically traverse TNTs to enter aged somatic cells. FTY720-treated donors effectively delivered mitochondria to untreated recipients, resulting in increased mitochondrial mass and ATP content in aged recipient GCs (Figure 4I-J, 4L-M). Western blot analysis further showed that recipient cells acquired elevated levels of cytoskeletal regulators and mitochondrial transport proteins, implicating a stable rewiring of intercellular infrastructure (Figure 4K, 4N). Given that cytoskeletal activation enhanced mitochondrial exchange, we further assessed metabolic changes induced by FTY720. Supplementary analyses confirmed improved mitochondrial function, reduced oxidative stress, and decreased ROS-induced cell death (Figure S2). These findings suggest that in aged granulosa cells with dysfunctional mitochondria, enforced cellular contact can facilitate selective transfer of healthier mitochondria from neighboring cells or through intraclonal redistribution, replacing damaged mitochondrial networks and restoring metabolic equilibrium.

### Three-Dimensional Microenvironment Enhances Intercellular Contact and Mitochondrial Function

**Figure 5:**
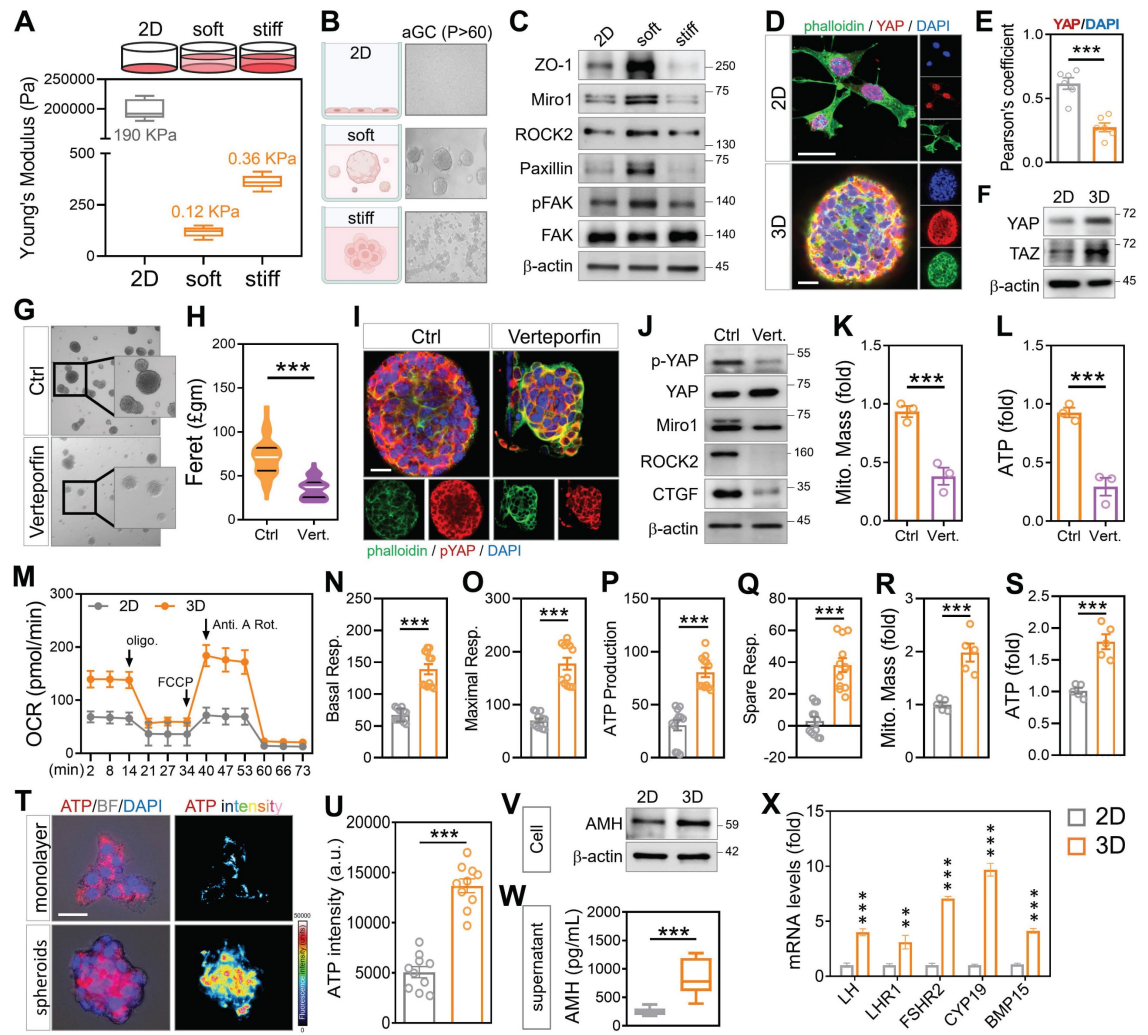
** Three-dimensional culture environments promote cell-cell interactions and support enhanced mitochondrial function.** (A) Schematic of hydrogel-based 3D culture with tunable stiffness. (B) Brightfield images showing HGL5 spheroid formation under soft matrix conditions. (C) Western blot of cytoskeletal markers across 2D, stiff, and soft conditions. (D-E) YAP/pYAP levels in different matrix conditions by western blot and quantification. (F) Western blot of YAP and TAZ levels. (G-H) Brightfield imaging and quantification of spheroid formation rate after YAP inhibition. (I) Immunostaining of pYAP distribution after YAP inhibition. (J) Western blot of cytoskeletal markers after YAP inhibition. (K-L) Mitochondrial mass and ATP measured by flow cytometry. (M-Q) OCR curves and respiratory parameter quantification in 2D and 3D-soft cultured aGCs. (R-S) Mitochondrial mass and ATP measured by flow cytometry. (T-U) Brightfield and merged fluorescence images with LUT-based fluorescence intensity analysis of patient-derived cumulus cells in 3D culture. (V-W) Western blot and ELISA quantification of AMH protein and secreted AMH in aGCs. (X) RT-qPCR analysis of hormone-related gene expression in aGCs cultured under 2D or 3D conditions. Scale bar = 20 µm. **p < 0.01 and ***p < 0.001.

Given that enforced cell-cell contact via cytoskeletal modulation enhanced mitochondrial exchange in aged GCs, we next asked whether this intercellular proximity could be achieved in a more physiologically relevant manner. To examine whether the microenvironment influences mitochondrial function in aGCs, we embedded long-term passaged HGL5 cells in 3D hydrogels with tunable stiffness. Under soft (0.12 kPa) ECM conditions, aGCs spontaneously formed multicellular spheroids, whereas cells in stiff (0.36 kPa) or 2D cultures remained spread and disconnected (Figure 5A-B). The metabolic enhancement we observed in 3D spheroids was not driven by an overall increase in transcriptional or translational load, but rather by improved mitochondrial efficiency and network coordination. Apoptosis, telomerase length, total RNA and protein were not increased, while the levels of genes important for mitochondrial function and reproductive endocrinology in aGC cells were elevated (Figure S3A-G). Western blot analysis revealed that soft matrix conditions activated cytoskeletal signaling and YAP pathway components, with increased ROCK2, Miro1, paxillin, and phosphorylated FAK (Figure 5C-E). Immunofluorescence confirmed that YAP localization shifted from the nucleus (in stiff 2D) to the cytoplasm (in soft 3D), consistent with YAP/TAZ pathway modulation (Figure 5F). To further assess whether YAP signaling contributes to spheroid assembly, we performed a reverse validation experiment using a YAP-TEAD inhibitor in aGCs. Brightfield imaging showed that YAP inhibitor-treated aGCs in soft 3D matrices formed fewer and smaller spheroids compared to untreated controls (Figure 5G), with quantitative analysis confirming a significant reduction in spheroid number and formation speed (Figure 5H). Notably, the resulting spheroids exhibited irregular, rougher edges rather than smooth, compact morphology, suggesting impaired cytoskeletal reorganization. Immunostaining showed reduced cytoplasmic accumulation of phosphorylated YAP (Figure 5I), and western blot analysis confirmed downregulation of cytoskeletal markers (Figure 5J).

Mitochondrial mass and ATP fluorescence intensity were both significantly lower in the YAP-inhibited group (Figure 5K-L and Figure S3H), despite exposure to the same physical matrix. To assess mitochondrial performance, Seahorse analysis showed that 3D-soft cultures had significantly higher oxygen consumption than 2D, with increases in basal respiration, maximal respiration, ATP production, and spare respiratory capacity (Figure 5M-Q). Flow cytometry further confirmed that soft 3D conditions enhanced mitochondrial mass (Figure 5R) and total ATP levels (Figure 5S). To determine whether combining cytoskeletal activation with 3D culture enhances recovery in aged granulosa cells, we applied FTY720 treatment under soft hydrogel conditions. This approach improved spheroid formation, ATP production, and mitochondrial membrane potential (Figure S3I-J). In contrast, disrupting the cytoskeleton with nocodazole led to smaller, irregular spheroids (Figure S3K-L), underscoring the importance of both physical contact and cytoskeletal integrity for metabolic restoration. To evaluate translational relevance, primary cumulus cells from a 39-year-old infertile patient were cultured in soft 3D hydrogels. These cells successfully formed spheroids and exhibited increased ATP intensity (Figure 5T-U). Notably, AMH protein expression was elevated under 3D conditions, as confirmed by western blot (Figure 5V), and ELISA analysis showed a significant increase in secreted AMH levels (Figure 5W). In addition, transcriptional profiling revealed upregulation of hormone-related markers such as FOXL2 and FSHR (Figure 5X), suggesting enhanced aged granulosa cell identity and follicular support capacity. Collectively, these findings indicate that mechanical cues can restore metabolic activity and endocrine function in clinically aged cumulus cells.

### Three-Dimensional Culture Induces Metabolic Reprogramming Toward Oxidative Phosphorylation

**Figure 6:**
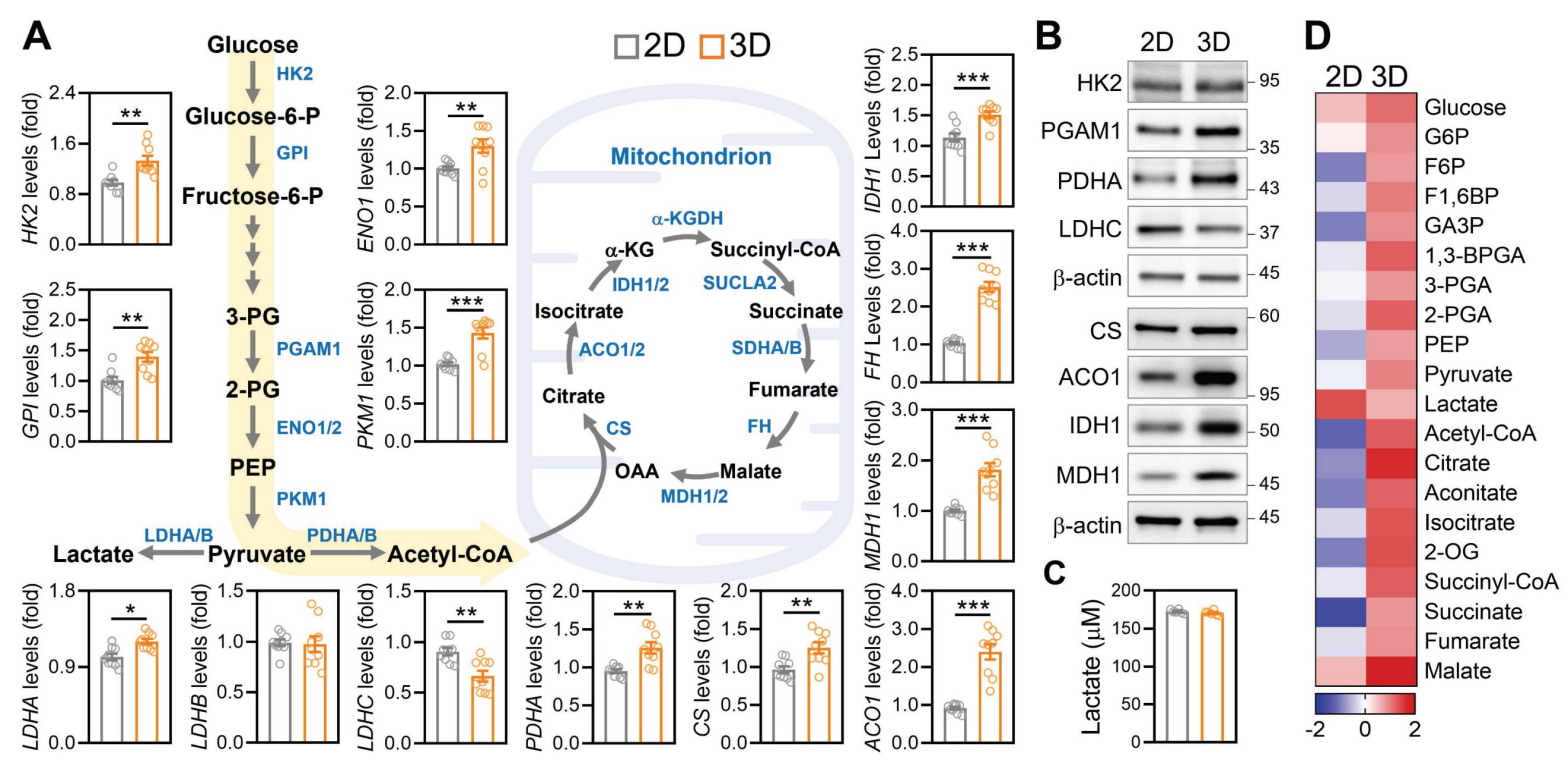
** Three-dimensional ECM environments promote metabolic reprogramming in aGCs.** (A) Gene expression of glycolytic and mitochondrial metabolic enzymes in aGCs cultured on 2D vs. 3D ECM conditions. Genes include *HK2*, *GPI*, *ENO1*, *PKM1*, *PDHA*, *CS*, *ACO1*, *IDH1*, *FH*, *MDH1*, and *LDH* isoforms. (B) Western blot analysis of HK2, PGAM1, LDHC, PDHA, CS, ACO1, IDH1, and MDH1 protein levels under 2D and 3D conditions. (C) Extracellular lactate concentration in culture medium from 2D versus 3D aGCs. (D) Heatmap representing relative metabolite abundance of central carbon metabolism intermediates in aGCs under 2D or 3D conditions. *p < 0.05, **p < 0.01, ***p < 0.001.

Given the observed improvements in mitochondrial mass, ATP production, and ovarian gene expression under 3D culture, we next investigated whether the ECM based microenvironment also restructured core metabolic pathways in aGCs. Transcriptomic analysis revealed that 3D cultured aGCs exhibited upregulated expression of key glycolytic enzymes, including *HK2, GPI, ENO1*, and *PKM1*, as well as mitochondrial metabolic regulators such as* PDHA1, ACO1, IDH1, FH, and MDH1*, relative to standard 2D culture (Figure 6A). This global upregulation suggested an overall enhancement in both glycolytic input and mitochondrial carbon oxidation. Western blotting further confirmed increased expression of HK2, PGAM1, LDHC, CS, ACO1, IDH1, and MDH1 proteins in 3D-cultured cells (Figure 6B), indicating that transcriptional activation translated into increased metabolic protein abundance. Importantly, these molecular changes were accompanied by a functional shift in energy production. Aged GCs maintained under 3D conditions secreted significantly less lactate compared to 2D controls (Figure 6C), suggesting reduced glycolytic overflow and improved metabolic efficiency. Consistent with this shift, targeted metabolomic profiling revealed elevated levels of tricarboxylic acid (TCA) cycle intermediates, including citrate, succinate, fumarate, and malate, in the 3D condition (Figure 6D), indicative of enhanced mitochondrial respiration. These data complement our earlier findings from mitochondrial transfer experiments (Figure 3), suggesting that both direct organelle supplementation and microenvironmental remodeling converge on a common metabolic endpoint: rejuvenation of mitochondrial energetics through rewiring of carbon flux.

### Cytoskeletal Activation Restores Folliculogenesis and Energy Metabolism in Aged Ovaries

**Figure 7:**
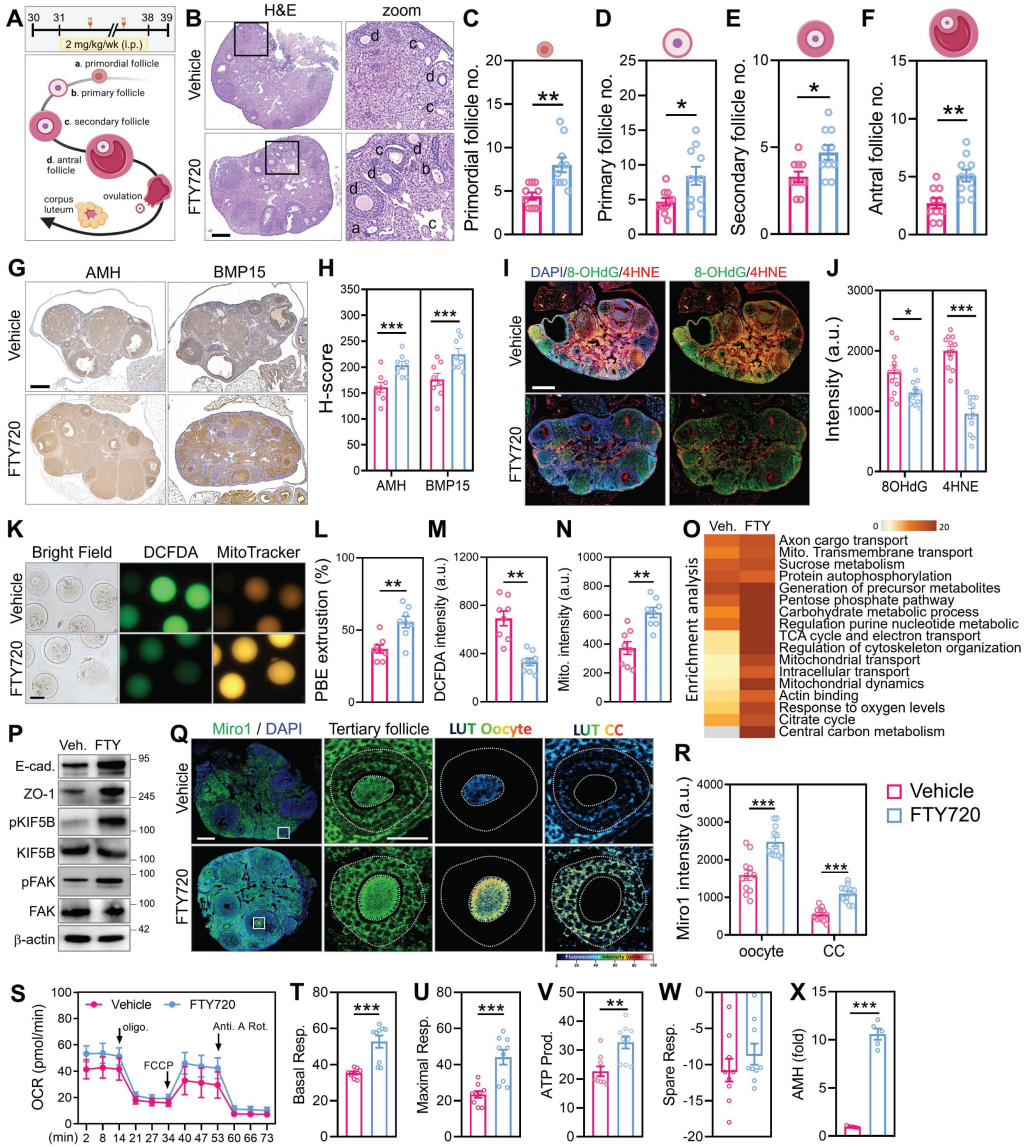
** FTY720 improves folliculogenesis, oocyte quality, and mitochondrial function in aged female mice.** (A) Schematic of FTY720 administration in reproductively aged mice. (B) H&E staining of ovarian sections from vehicle- and FTY720-treated mice. (C-F) Quantification of follicle numbers at different stages: primordial (C), primary (D), secondary (E), and antral (F). (G-H) Immunohistochemical staining and quantitative analysis of AMH and BMP15 expression in ovarian tissue sections. (I-J) Immunofluorescence staining and quantitative analysis of oxidative stress markers 8-OHdG and 4-HNE. (K) Fluorescence imaging of ovulated MII oocytes stained with DCFDA and MitoTracker. (L-N) Quantification of (L) polar body extrusion number, (M) DCFDA fluorescence intensity, and (N) MitoTracker fluorescence. (O) RNA-sequencing and pathway enrichment analysis of ovarian tissues. (P) Western blot analysis of mitochondrial trafficking and biogenesis markers in ovaries from aged mice treated with vehicle or FTY720. (Q) Representative immunofluorescence images of ovarian sections stained for Miro1 and DAPI in tertiary follicles. Pseudocolor LUT was applied to quantify Miro1 intensity. (R) Quantification of Miro1 fluorescence intensity in oocytes and cumulus cells from (Q). (S) OCR tracing of whole ovaries measured by Seahorse assay. (T-W) Quantification of basal respiration (T), maximal respiration (U), ATP-linked respiration (V), and spare respiratory capacity (W). (X) Serum AMH levels measured by ELISA. (K) Scale bar = 20 µm. (B, I, Q) Scale bar = 200 µm. *p < 0.05, **p < 0.01, ***p < 0.001.

To assess the *in vivo* efficacy of cytoskeletal stimulation on ovarian aging, reproductively aged female mice were treated with FTY720 for 8 weeks (Figure 7A). Histological analysis revealed preserved ovarian morphology in the FTY720 group, with higher numbers of follicles across developmental stages compared to vehicle-treated controls (Figure 7B). Quantification showed increased counts of primordial, primary, secondary, and antral follicles (Figure 7C-F). To evaluate follicle quality, we performed immunohistochemical staining for AMH and BMP15, two somatic and oocyte-derived markers of follicular integrity (Figure 7G). Signal intensities for both markers were elevated in FTY720-treated ovaries (Figure 7H), indicating preserved granulosa-oocyte signaling. To assess oxidative stress levels, ovarian sections were stained by immunofluorescence for 8-OHdG and 4-HNE. These markers of DNA and lipid oxidative damage were reduced in FTY720-treated mice (Figure 7I), with corresponding decreases in fluorescence intensity (Figure 7J). To assess mitochondrial function in ovulated oocytes, we collected MII-stage oocytes from aged mice and stained them with MitoTracker and DCFDA (Figure 7K). Following FTY720 treatment, the number of MII oocytes increased (Figure 7L). These oocytes exhibited stronger MitoTracker fluorescence and reduced DCFDA signal compared to controls, indicating improved mitochondrial activity and lower intracellular ROS levels (Figure 7M-N). Transcriptomic profiling of ovaries by RNA-seq revealed differential expression of genes involved in mitochondrial transport, cytoskeletal remodeling, and follicle development (Figure 7O).

To validate the effect of FTY720 on the ovarian microenvironment, we examined cell-cell contact and cytoskeletal signaling markers by western blot. FTY720-treated ovaries showed increased expression of E-cadherin and ZO-1, indicating enhanced cell-cell adhesion. In addition, phosphorylation levels of KIF5B and FAK were elevated, suggesting improved cytoskeletal activation and organelle trafficking capacity (Figure 7P). These findings support the notion that FTY720 promotes not only mitochondrial dynamics but also structural remodeling of the ovarian niche. Immunofluorescence further confirmed Miro1 upregulation in both oocytes and surrounding cumulus cells (Figure 7Q-R). Seahorse analysis revealed increased mitochondrial respiration in the FTY720 group, as shown by elevated basal and maximal OCR, ATP-linked respiration, and spare respiratory capacity (Figure 7S-W). Finally, serum AMH levels measured by ELISA were higher in treated animals (Figure 7X), suggesting enhanced follicular endocrine output following cytoskeletal stimulation. We also analyzed clinical cumulus cell samples from a 39-year-old infertile patient to validate the relevance of this mechanism. After FTY720 treatment, brightfield and MitoTracker imaging revealed TNT formation and visible mitochondrial transfer between cells (Figure S4A). RNA-seq analysis showed widespread transcriptional changes, with upregulation of genes involved in mitochondrial transport, cytoskeletal remodeling, and oxidative phosphorylation (Figure S4B-C). Moreover, the FTY720-induced increases in mitochondrial fusion and transport proteins were abolished by co-treatment with nocodazole, confirming that this process depends on intact cytoskeletal structures (Figure S4D). To further assess functional improvement* in vivo*, we analyzed markers of ovarian reserve and senescence. Quantitative analysis showed that ovarian AMH expression was significantly elevated in FTY720-treated aged mice compared to vehicle controls (Figure S4E), suggesting enhanced follicular function. In contrast, the expression of the senescence marker *p21* remained unchanged (Figure S4F), indicating that FTY720 improved ovarian output without altering global senescence status.

## Discussion

Mitochondrial dysfunction is widely recognized as a central hallmark of reproductive aging. In both germ cells and their surrounding somatic support, namely GCs and CCs, aging leads to a decline in mitochondrial membrane potential, ATP output, and mitochondrial quality control, along with an increase in oxidative stress and ROS accumulation 6, 9, 30. These changes compromise oocyte developmental competence and drive the progressive decline in female fertility. Traditional interventions for counteracting mitochondrial insufficiency, such as coenzyme Q10 supplementation or direct mitochondrial replacement therapies, have offered partial improvement in animal models but remain limited in scope, scalability, or regulatory feasibility in humans 31, 32.

Our study proposes an alternative framework by demonstrating that the aging ovarian microenvironment can be metabolically and structurally reprogrammed through somatic cell derived mitochondrial transfer. Specifically, we show that donor somatic cells, including granulosa like cells, are capable of transferring mitochondria into aged oocytes via cytoskeletal projections that traverse the zona pellucida. This phenomenon represents a physiological, cell directed mechanism of intercellular organelle sharing, one that has been observed in other contexts such as neuronal and immune cell regeneration 18, 19, 21, but has not previously been implicated in the ovarian follicle. The mechanistic foundation of this transfer process lies in cytoskeletal remodeling. As we demonstrate, partial digestion of the zona pellucida was required to enable physical connection between donor cells and the oocyte membrane. Once contact was established, donor cells extended transzonal like filopodia, thin actin rich projections analogous to tunneling nanotubes, through which RFP labeled mitochondria were trafficked. These projections bear resemblance to gap junction associated transzonal projections in healthy preovulatory follicles, which decline with age 5, 12, 15. By reinstating physical connectivity through engineered contact, we effectively restored a key dimension of oocyte somatic communication.

This approach differs substantially from mitochondrial injection techniques. Instead of delivering mitochondria through invasive means, our strategy allows for endogenous cell to cell communication and continuous bioenergetic support. In contrast to ooplasmic transfer, which often results in mitochondrial heteroplasmy and has triggered ethical and regulatory scrutiny 10, 17, 33, our approach uses autologous or patient matched donor cells, eliminating the risk of intergenomic conflict. Moreover, the transferred mitochondria integrate naturally into the oocyte cytoplasm and contribute to improved membrane potential, mitochondrial abundance, and functional readouts, such as ROS clearance and ATP levels. From a translational standpoint, this discovery opens compelling new avenues in reproductive medicine. Women experiencing age related infertility, diminished ovarian reserve, or those undergoing chemotherapy may benefit from *ex vivo* or* in situ* rejuvenation of their oocytes through cell mediated mitochondrial supplementation. For example, mesenchymal stem cells or other autologous somatic sources could be expanded, differentiated into granulosa like cells, and co cultured with the patient's oocytes in* in vitro* fertilization settings. Given that our study demonstrates significant improvements in fertilization rates, blastocyst development, and even live births in aged murine models, the potential for clinical adaptation is strong.

Our findings are also relevant in the context of existing rejuvenation approaches. Nicotinamide adenine dinucleotide precursors, such as nicotinamide mononucleotide, have been shown to improve oocyte function in aged animals by enhancing the activity of sirtuins and mitochondrial enzymes 9, 30, 34. However, these systemic agents rely on residual mitochondrial functionality and do not address cases where mitochondrial number or integrity is severely diminished. Similarly, caloric restriction mimetics and antioxidants can modulate redox homeostasis, but their effect sizes are variable, and long-term data in humans are lacking 32, 35. Our strategy, in contrast, delivers whole, functional mitochondria from supportive cells directly into the oocyte, essentially replenishing the cellular power source itself. The broader significance of mitochondrial sharing extends beyond fertility. Recent studies have shown that mitochondria can act as signaling organelles, modulating nuclear transcription, epigenetic states, and apoptotic thresholds 36-38. Thus, mitochondrial donation may not only restore energetic function but also reprogram cell fate decisions in aging oocytes. This could explain the downstream improvements we observed in embryo morphology and viability, which may be due in part to re-established mitochondrial nuclear coordination.

In aged granulosa cells, conventional 2D culture conditions induced a metabolic shift toward glycolysis, accompanied by increased YAP activity and fragmented mitochondrial networks. This shift is likely due to the absence of physiologic ECM cues and altered mechanotransduction inherent to flat, rigid substrates. Prior studies have shown that substrate stiffness and dimensionality profoundly influence cellular metabolism by modulating cytoskeletal tension and YAP/TAZ signaling 39-41. In contrast, culturing aged granulosa cells within soft 3D hydrogels reinstated a supportive matrix environment, reduced nuclear YAP localization, and restored mitochondrial oxidative phosphorylation. These findings underscore the pivotal role of ECM-mediated mechanical signaling in governing the metabolic state of aging granulosa cells.

Beyond mechanistic insights, our findings also inform future directions in reproductive tissue engineering. Integrating mitochondrial sharing into three-dimensional follicular organoid systems or bioengineered ovarian scaffolds could enhance *in vitro* folliculogenesis and support oocyte maturation outside the body. Such platforms may serve not only for fertility restoration, but also as physiologically relevant models for studying ovarian aging in high-throughput formats. Looking forward, several translational avenues emerge. First, *in vivo* validation through orthotopic transplantation of mitochondria-donor cells into aged ovaries could confirm therapeutic efficacy without requiring *ex vivo* manipulation. Second, the development of small molecule enhancers that target mitochondrial trafficking pathways, such as those involved in actin polymerization or mitofusin mediated fusion, may offer a noninvasive strategy to promote organelle sharing *in vivo*. Third, combining mitochondrial transfer with other rejuvenation strategies, including NAD⁺ boosters, extracellular vesicle-based signaling, or cytoskeletal stabilizers, may further optimize regenerative outcomes.

## Conclusion

Our findings reveal that intercellular mitochondrial sharing, facilitated by structural remodeling, represents a powerful and underappreciated mechanism to rescue oocyte quality in the aging ovary. This work reframes the ovarian niche as an active participant in cellular rejuvenation, capable of being therapeutically reprogrammed. By leveraging intrinsic support mechanisms within the follicular microenvironment, we lay the groundwork for a new class of regenerative reproductive therapies aimed at extending fertility and improving women's reproductive autonomy.

## Supplementary Material

Supplementary figures and tables.
