# Supplementary material for: Reprogramming the aging ovarian microenvironment via mitochondrial sharing and structural remodeling
Source: Theranostics. 2025 Aug 16;15(17):9279–93. doi: 10.7150/thno.119957 (PMC12439467; doi:10.7150/thno.119957)
Supplement: Supplementary file 1 — Supplementary figures and tables. [file thnov15p9279s1.pdf]

Table S1. Primer sequences designed for RT-PCR

Primers	Forward	Reverse
HK2	F-TCCGTAACATTCTCATCGATTTCA	R-TGTCTTGAGCCGCTCTGAGAT
GPI	F-GACCCAGCACCCCATACG	R-CAAGAAGTTGGCCAGGAGGAT
ENO1	F-TGGGAAAGCTGGCTACACTGA	R-CTCGGAGGCCGCTACGT
ENO2	F-AAGGCTGGCTACACGGAAAA	R-CGATAAAACTCTGAGGCAGCAA
PKM	F-TCTGAGCGGTCTTTGCTAGTGA	R-TGACATAATGCTCCCCTTTTGG
LDHA	F-GAAGCGGTTGCAATCTGGAT	R-GGTGAACTCCCAGCCTTTCC
LDHB	F-GGGAACATGGCGACTCAAGT	R-GAGAAACACCTGCCACATTCAC
LDHC	F-GGGCTATTGGACTGTCTGTGATG	R-TGGGTGCACTCTCCTAAGATTTTT
PDHA1	F-ACCCCACAGACCATCTCATCA	R-CCCCGGGTGAAAGTAAAGC
PDHB	F-AACTGTGGTTTCCCATTCAAGAC	R-TTAGATAGCACTGCTGCAGCTTCT
CS	F-TCTGGAGCCGAGCCTTAGG	R-GACCCTCTGTGCTCATGGACTT
MDH1	F-GCTGTCATCAAGGCTCGAAAAC	R-GGTCACAGATGGCTTTTGCA
MDH2	F-TGCCCGGAAGCCATGAT	R-GACTCGAGCTGGATCCAAACC
SUCLA2	F-GCA AGA AGCTGG TGT CTC CGT T	R-GGC AAC ACC AAG CTT TGC A
KGDH	F-TGC TCG GCA ATT CAG TCA TC	R-GCC AGT GTG CCA TCG CTT A
IDH1	F-CGG AAC CCA AAA GGT GAC AT	R-TGG CAA CAC CAC CAC CTT CT
IDH2	F-CCT GGC GGG CTG CAT	R-GGA AGT GCT CGT TCA GCT TCA
ACO1	F-GGT TTG ACG TGG TGG GCT AT	R-TCA GGT AAA GGC CCA CTG TTG
ACO2	F-TCA ACC CAG AGA CCG ACT ACC T	R-GAG CCT CCA GCC TGA ACT TCT
LH	F-ATC CTC ATC TTC ACC GAT TTC AC	R-GGC AGC TGA GAT GGC AAA A
LHR1	F-CAT TCA ATG GGA CGA CAC TG	R-GCC TCC AGG AGA TTG ACA AA
FSHR2	F-CTT TTG CAG CTG CCC TCT TT	R-GGC AGA TGC TCA CCT TCA TGT
CYP19	F-GGA AAA ATC CGC ACA CAC AA	R-CTC CCC ACC TCC CAA CTC A
BMP15	F-GGA GTT GTA CCG GCG TTC AG	R-CCC AAT GGT GCG GTT CTC T
P16	F-TGTGTTGGAGTTTTCTGGAGTGA	R-CAAGAAATGCCCACATGAATGT
P21	F-TGGAGACTCTCAGGGTCGAAAA	R-GCGTTTGGAGTGGTAGAAATCTG
RNU6-1	F-CTCGCTTCGGCAGCACATATACT	R-ACGCTTCACGAATTTGCGTGT C

Table S2. 1st antibodies for immunoblotting, and immunofluorescence

Antigen	Host	Cat.	Type	Source
DRP1	Mouse	ab56788	monoclonal	Abcam
DRP1 Ser616	Rabbit	3455	monoclonal	CellSignaling
FIS1	Rabbit	GTX111010	polyclonal	Genetex
MFN1	Rabbit	GTX133351	polyclonal	Genetex
MFN2	Mouse	ab56889	monoclonal	Abcam
OPA1	Rabbit	GTX129917	polyclonal	Genetex
HK2	Rabbit	2867	polyclonal	CellSignaling
PGAM1	Rabbit	NBP1-49532	polyclonal	NOVUS
PDHA	Rabbit	A1895	polyclonal	ABclonal
PDHB	Rabbit	A6943	polyclonal	ABclonal
LDHA	Rabbit	A1146	polyclonal	ABclonal
LDHB	Rabbit	A7625	polyclonal	ABclonal
LDHC	Rabbit	A15003	polyclonal	ABclonal
CS	Rabbit	A5713	polyclonal	ABclonal
ACO1	Rabbit	A7867	polyclonal	ABclonal
ACO2	Rabbit	A3716	polyclonal	ABclonal
IDH1	Rabbit	A2169	polyclonal	ABclonal
IDH2	Rabbit	A7190	polyclonal	ABclonal
MDH1	Rabbit	A7563	polyclonal	ABclonal
SDHB	Rabbit	A10821	polyclonal	ABclonal
FH	Rabbit	A5688	polyclonal	ABclonal
E-Cad	Rabbit	20874-1-AP	polyclonal	Proteintech
Paxillin	Rabbit	2542	polyclonal	CellSignaling
ZO-1	Rabbit	5406	polyclonal	CellSignaling
FAK	Rabbit	3285	polyclonal	CellSignaling
pFAK	Rabbit	3283	polyclonal	CellSignaling
KIF5B	Rabbit	A15284	polyclonal	ABclonal
pKIF5B	Rabbit	AF7447	polyclonal	Affbiotech
Miro1	Rabbit	GTX31938	polyclonal	Genetex
ROCK2	Rabbit	A2395	polyclonal	ABclonal
CTGF	Rabbit	A11456	polyclonal	ABclonal
AMH	Rabbit	GTX129593	polyclonal	Genetex
YAP	Mouse	sc-101199	monoclonal	Santa cruz
pYAP	Rabbit	29018-1-AP	polyclonal	Proteintech
TAZ	Rabbit	72804	polyclonal	CellSignaling
BMP15	Rabbit	18982-1-AP	polyclonal	Proteintech
8-OHdG	Mouse	GTX41980	monoclonal	Genetex
4-HNE	Rabbit	GTX01087	polyclonal	Genetex
Beta actin	Rabbit	GTX109639	polyclonal	Genetex
Alpha tubulin	Mouse	GTX628802	monoclonal	Genetex

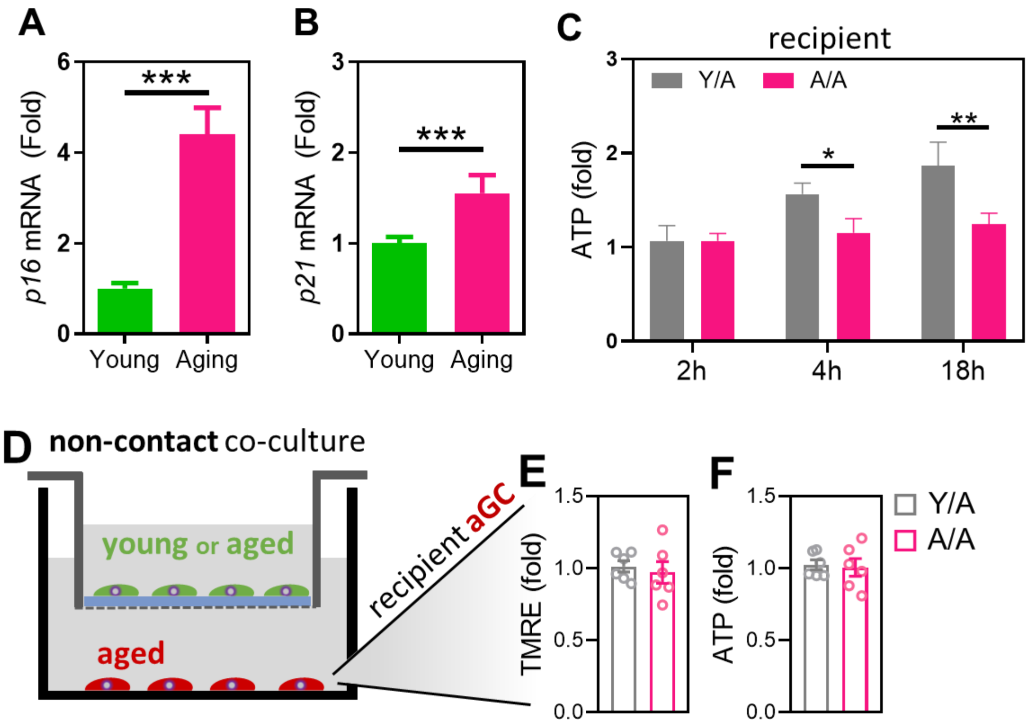

Figure S1. Characterization of aging status and contact-dependent mitochondrial rescue in granulosa cells. (A–B) Quantitative PCR analysis of senescence markers *p16* and *p21* mRNA levels in yGCs and aGCs. (C) Time-course quantification of intracellular ATP levels in aged recipient cells after co-culture with yGCs. (D) Schematic of transwell-based indirect co-culture system preventing direct contact between donor and recipient cells. (E–F) Flow cytometric analysis of mitochondrial membrane potential (TMRE) and intracellular ATP intensity in recipient aGCs. *$p < 0.05$, **$p < 0.01$, ***$p < 0.001$.

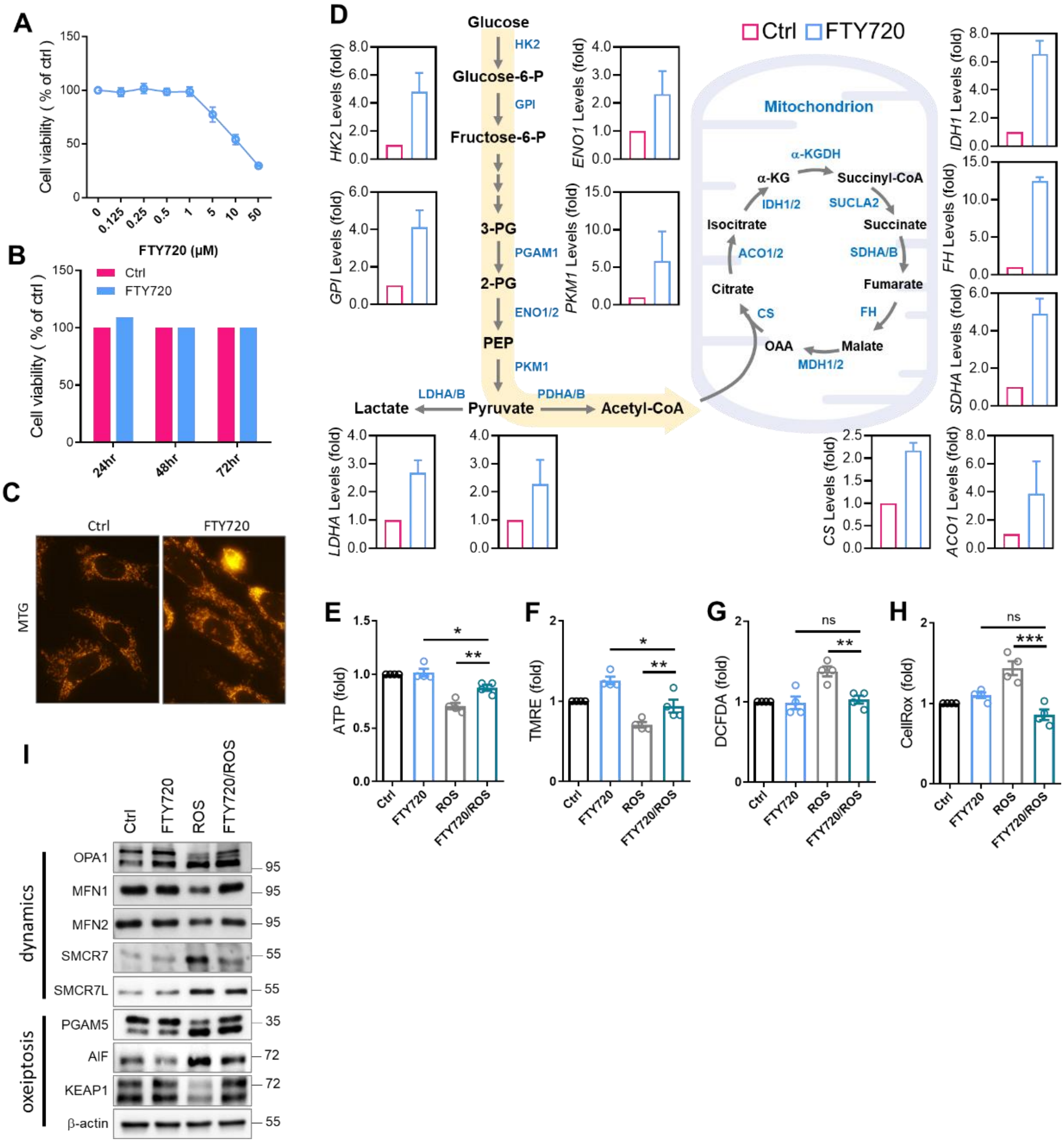

Figure S2. FTY720 prevents ROS-induced oxeiptosis in aGCs. (A) Cell viability assay of aGCs treated with increasing concentrations of FTY720 (0.1–10 μM, 24 h). (B) Time-course viability assessment of aGCs treated with 1 μM FTY720 at 24, 48, and 72 h. (C) Representative fluorescence images of mitochondrial morphology visualized by MitoTracker staining after FTY720 treatment (1 μM). (D) qPCR quantification of metabolic gene expression (glycolytic and mitochondrial pathways). (E–H) Fluorescence-based quantification of intracellular ATP (E), mitochondrial membrane potential via TMRE (F), intracellular ROS by DCFDA (G), and oxidative stress levels using cellROX (H) in vehicle- and FTY720-treated cells. (I) Western blot analysis of mitochondrial dynamics proteins and oxeiptosis-related markers in aGCs following treatment with FTY720. *p < 0.05, **p < 0.01, ***p < 0.001.

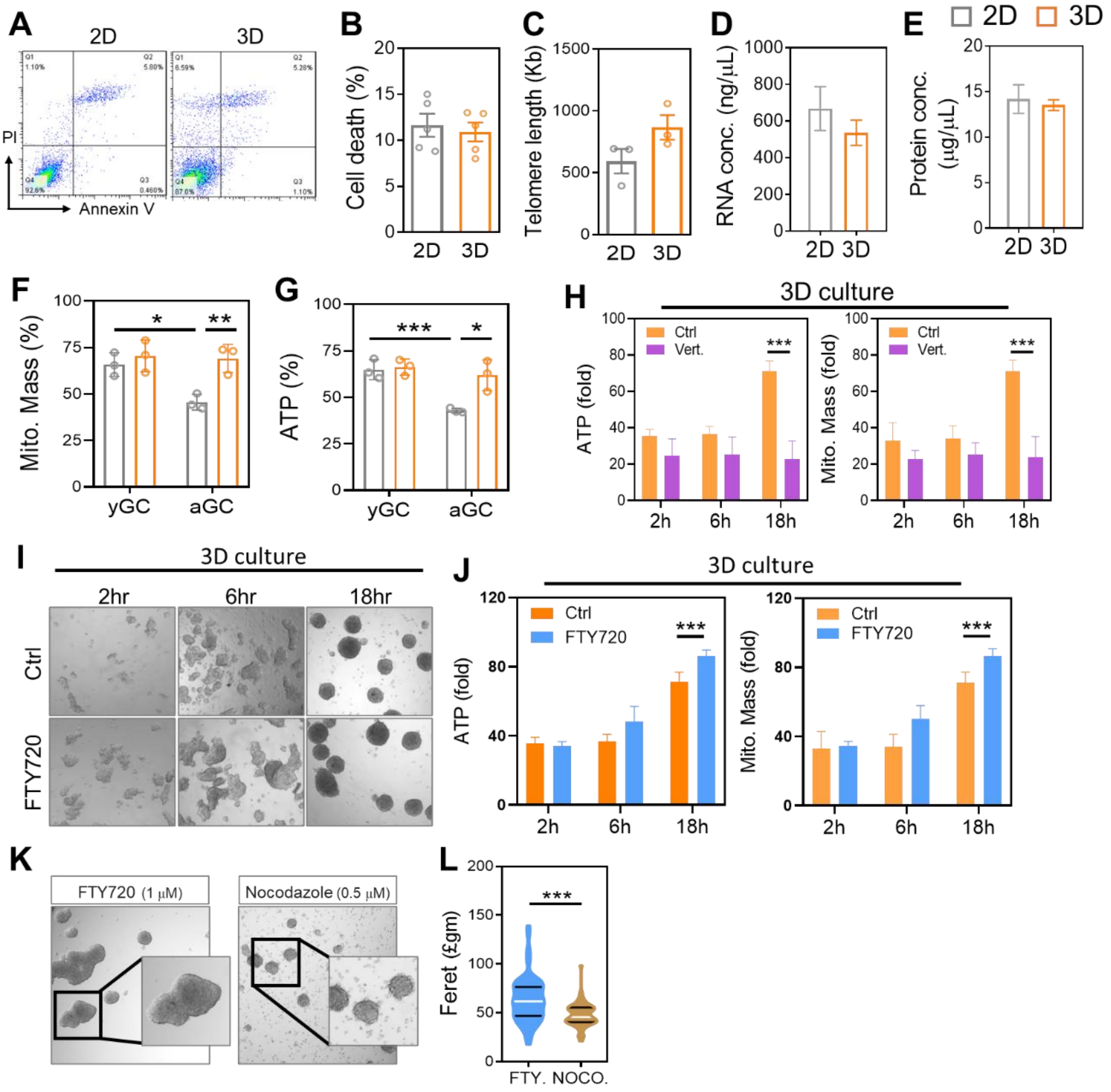

Figure S3. 3D spheroid formation does not induce cell death or biosynthetic burden and requires intact cytoskeletal signaling. (A–B) Annexin V/PI staining of aGCs cultured in 2D or soft 3D conditions. (C) Relative telomere length measurement in aGCs under 2D and soft 3D culture. (D–E) Quantification of total intracellular RNA and protein content in aGCs cultured under 2D and soft 3D conditions. (F–G) Flow cytometry analysis of ATP levels and mitochondrial mass in 2D or 3D. (H) Quantification of mitochondrial mass and ATP fluorescence intensity in spheroids. Both parameters were significantly reduced in the YAP inhibitor group compared to control. (I) Brightfield images of aGCs cultured in 3D hydrogels with or without FTY720 for 2, 6, and 18 hours. (J) Fluorescence-based quantification of intracellular ATP levels and mitochondrial mass at the indicated time points. Both parameters increased over time, with significantly higher values observed in the FTY720 group at 18 hours. (K–L) Brightfield imaging of spheroids formed from aGCs treated with FTY720 or FTY720 + nocodazole. $^{*}p < 0.05$, $^{**}p < 0.01$, $^{***}p < 0.001$.

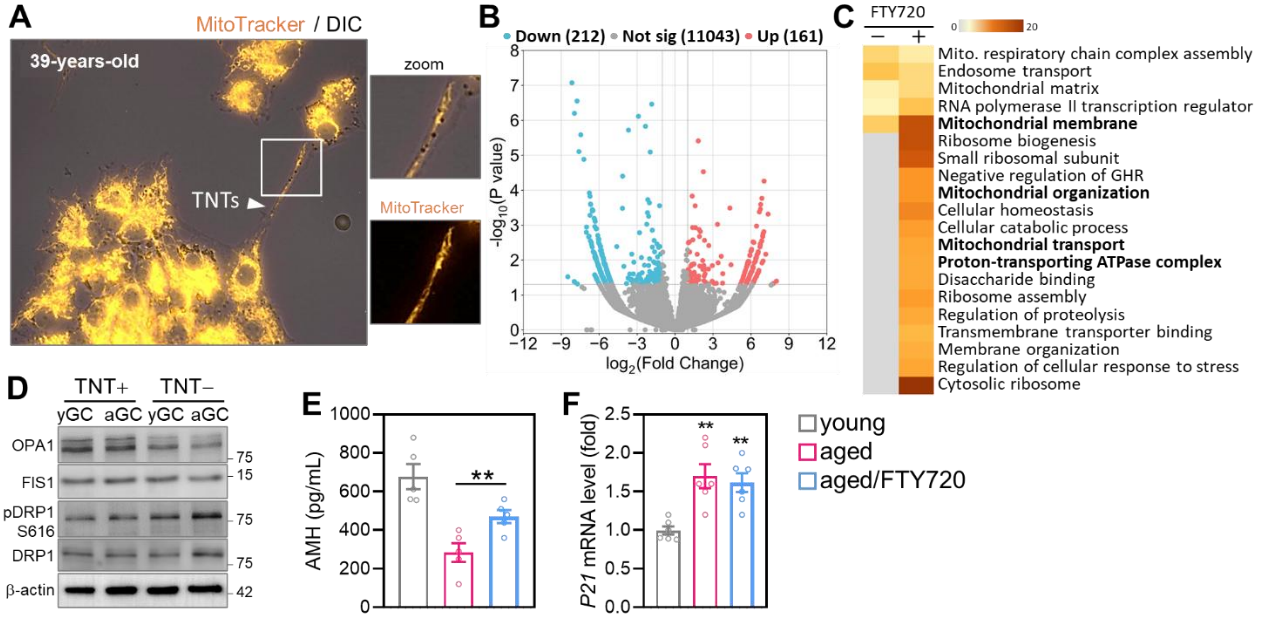

Figure S4. Cytoskeletal stimulation reprograms mitochondrial trafficking in patient-derived granulosa cells. (A) MitoTracker-labeled mitochondria visualized trafficking through TNTs in granulosa cells from a 39-year-old patient post-FTY720 treatment. (B) Volcano plot of RNA-seq data from treated vs. control cells. (C) Heatmap of top enriched pathways, including mitochondrial dynamics and cytoskeletal remodeling. (D) Western blot shows that nocodazole blocks FTY720-induced upregulation of mitochondrial transport and fusion proteins. (E) Quantification of AMH protein expression in ovaries from the same groups, indicating improvement in ovarian reserve following FTY720 treatment. (F) Quantification of p21 expression showing no significant change in senescence marker levels across groups. **p < 0.01.
